# The Impact of Virtual Reality Immersion on Learning Outcomes: A Comparative Study of Declarative and Procedural Knowledge Acquisition

**DOI:** 10.3390/bs15101322

**Published:** 2025-09-26

**Authors:** Nengbao Yu, Wenya Shi, Wei Dong, Renying Kang

**Affiliations:** 1 School of Humanities, Tiangong University, Tianjin 300387, China; ynb@tiangong.edu.cn; 2 School of Education, Tianjin University, Tianjin 300072, China; swy16@tju.edu.cn (W.S.); kry_1026311460@tju.edu.cn (R.K.)

**Keywords:** virtual reality, immersion, declarative knowledge, procedural knowledge, learning effectiveness

## Abstract

The potential of Virtual Reality (VR) in enhancing learning and training is being widely explored. The relationship of immersion, as one of the core technical features of VR, with knowledge types has not been fully explored. This study aims to investigate how VR immersion levels (high vs. low) affect the acquisition of declarative and procedural knowledge, as well as related cognitive and affective factors. A 2 × 2 mixed design was adopted, with 64 college students who had no VR experience and no background in professional medical knowledge being randomly assigned to either a high-immersion group (using HTC Vive Pro headsets) or a low-immersion group (using desktop monitors). Participants completed learning tasks on thyroid and related diseases (declarative knowledge) and cardiopulmonary resuscitation (procedural knowledge), followed by knowledge tests and self-report questionnaires to measure presence, motivation, self-efficacy, cognitive load, and emotional states. Results showed that high immersion significantly improved learning outcomes for both types of knowledge with large effect sizes. In both knowledge domains, high immersion also enhanced presence, intrinsic motivation, self-efficacy, and positive emotions. However, cognitive load was reduced only for declarative knowledge, and no significant effects were observed for self-regulation. These findings highlight the differential impact of VR immersion on knowledge acquisition and provide insights for optimizing VR-based educational interventions.

## 1. Introduction

Virtual Reality (VR) can generate three-dimensional environments through computer simulation and, combined with the use of specialized devices, enable users to have a strong sense of immersion and lifelike interactive experience (Combalia et al., 2024). Immersion, Interaction, and Imagination are the three fundamental characteristics of VR technology (Mihelj et al., 2014). Immersion describes the objective property of a specific system to occlude the physical world while presenting a vivid virtual environment (Slater & Wilbur, 1997; Cummings & Bailenson, 2015), rather than being a product of individual subjective experience (Witmer & Singer, 1998). The stronger the high-fidelity characteristics of the display and tracking functions provided by the system across various perceptual modalities, and the higher the ability to reproduce real-world sensory experiences, the stronger the immersion. Based on differences in immersion levels, VR systems can be classified into Desktop Virtual Reality (DVR) and Immersive Virtual Reality (IVR) (Makransky et al., 2019). DVR relies on traditional input media such as keyboards and mice for interaction with computer screens, rarely or almost not isolating the physical environment, and only provides low-immersion experiences; IVR typically allows users to access via head-mounted displays or cave-style automatic virtual environments, effectively isolating the physical environment through multimodal sensory deprivation and providing high-immersion experiences.

Virtual Reality (VR) is being widely explored for its potential in enhancing learning and training due to its characteristics of situationality, interactivity, and embodiment (Mayer et al., 2023). As a core feature of VR, immersion receives extensive attention in research. These studies present the same learning content in forms such as IVR, DVR, or video, and investigate the effectiveness of immersive learning environments through media comparison methods. Previous research shows that high-immersion environments endow content and interaction with authenticity, which can strengthen learners’ sense of presence and flow (Guerra-Tamez, 2023), increase enjoyment and interest (Makransky & Mayer, 2022), and thereby foster stronger learning motivation., thereby increasing enjoyment and interest to foster stronger learning motivation (Parong & Mayer, 2018). Additionally, such settings not only deepen the comprehension and retention of complex concepts (Lui et al., 2020; Bagher et al., 2020), and promote generative processing and enhance cognitive transfer (Passig et al., 2016) so that knowledge can be flexibly applied to novel virtual or real world contexts, but also effectively improve cognitive skills (e.g., problem solving; Kozhevnikov et al., 2013), psychomotor skills (e.g., surgical procedures; Alaker et al., 2016), and affective skills through authentic scenario simulation. However, some studies show that high-immersion environments, due to their excessively rich information elements, result in high memory workload, leading to task performance and learning outcomes comparable to or even worse than low-immersion environments (Delgado & Mayer, 2025; Webster, 2016; Parong & Mayer, 2018; Makransky et al., 2019).

The inconsistency of the above conclusions may be partly related to the control of immersion in experiments (Makransky & Mayer, 2022) and may also be moderated by knowledge types (Parong & Mayer, 2021). Existing multimedia research shows that there is a certain compatibility relationship between media and knowledge types. Most of the learning materials presented in previous studies belong to declarative knowledge. However, declarative knowledge and procedural knowledge are distinct. The former addresses “what” and is represented as chunks. Its acquisition entails encoding, consolidation, and retrieval. The latter addresses “how” and is represented as production rules that must be compiled, strengthened, and automated through repeated practice (Anderson, 1982). An empirical study found that declarative knowledge and procedural knowledge have different effects in video learning (Hong et al., 2018), yet their applicability to virtual reality remains to be verified. Their responses to high-immersion VR are likely to differ. On the one hand, VR enriches factual encoding by integrating multisensory cues and embedding them in vivid contexts, thereby fostering deep processing of declarative memories. When simulated details are irrelevant to the learning objectives, however, such redundancy diverts attention and consumes cognitive resources, weakening the focus on core facts. On the other hand, VR optimizes the acquisition of procedural knowledge by presenting realistic situations, enabling safe rehearsal, and creating strong situational memories that support repeated condition-action coupling.

Current research on VR learning effectiveness has mostly focused on the impact of technical features (such as interaction, immersion, etc.) while neglecting attention to the nature of learning materials. The compatible knowledge types of immersion, as an important technical characteristic, remain under-explored. To address this, this study combines a knowledge-classification framework with a controlled-immersion design to examine how different immersion levels influence learning outcomes and learning processes for different knowledge types, thereby delineating the boundary conditions under which VR immersion enhances knowledge acquisition. Two research questions are posed: (1) Do learning outcomes differ significantly when learners study declarative versus procedural knowledge under different immersion levels? (2) Do learners differ significantly in affective variables (presence, self-efficacy, intrinsic motivation, emotion) and cognitive variables (self-regulation, cognitive load) when studying declarative versus procedural knowledge under different immersion levels?

## 2. Literature Review and Hypotheses

### 2.1. The Impact of VR Immersion on Learning Effectiveness

VR Immersion is one of the key features that distinguish IVR from DVR and non-immersive media such as videos and PowerPoint presentations (Makransky & Petersen, 2021). Currently, there is no consensus on the impact of VR Immersion on learning effectiveness. Some studies found that applying high-immersion environments to instruction has the potential to enhance learning outcomes. For example, Calvert and Abadia (2020) used the Kokoda Campaign in Australian history as teaching content and found that the IVR group performed better in learning compared to the 360° video learning environment. Similarly, Makransky and Mayer (2022) explored the application of IVR in climate change education and found that students who used high-immersion media (IVR) for virtual field trips performed better in both immediate and delayed test scores than those using low-immersion media (2D videos). Additionally, studies have shown that IVR is more suitable than PC for training doctors in delivering bad news, helping improve the quality and efficiency of social skills training (Ochs et al., 2019).

However, some studies found that high-immersion learning environments do not provide significant advantages and may even lead to poorer learning performance (Buttussi & Chittaro, 2018; Stepan et al., 2017; Parong & Mayer, 2018). For example, Klingenberg et al. (2020) examined the differences in learning the electron transport chain between undergraduate biochemistry students using IVR and DVR, and found no significant difference in academic performance between the two groups when only media conditions were considered. Similarly, Parong and Mayer (2018) showed that when learning biological knowledge, the IVR group showed poorer learning performance and higher cognitive load compared to the DVR group.

### 2.2. Learning of Declarative and Procedural Knowledge in VR

According to Anderson’s ACT-R (Adaptive Control of Thought-Rational) theory (Anderson, 1982), knowledge is classified into declarative and procedural forms based on their representational modes, reflecting distinct mechanisms for information storage and processing in the human cognitive system. Previous multimedia studies have shown that knowledge types and presentation methods affect learners’ learning effectiveness (Mayer & Anderson, 1991; Hong et al., 2018), indicating that the nature of the content itself should be considered when selecting media. For example, Hong et al. (2018) found that declarative and procedural knowledge differ in learning outcomes and cognitive load when studied in a video-based environment.

Research on immersive VR also indicates that learning outcomes are influenced by the type of knowledge being taught. Webster (2016) reported that IVR-based instruction yielded greater declarative knowledge gains than traditional teaching, whereas Buttussi and Chittaro (2018) found no significant difference between immersive and desktop VR in the acquisition or retention of procedural aviation-safety knowledge. These results suggest that high-immersion environments are not necessarily ideal media for knowledge transmission, largely depending on the nature of the knowledge being conveyed and the design approach. Mayer (2020) also noted that in media comparison studies involving immersion, it is necessary to identify learning materials or task types suitable for immersive environments to help optimize instructional design and improve learning effectiveness.

However, few studies adopt knowledge classification frameworks to distinguish different learning contents and compare the impact of immersive environments on learning effectiveness across knowledge types. Existing studies mostly focused on a single type of knowledge in specific domains/disciplines (Lui et al., 2020; Richards & Taylor, 2015; Kozhevnikov et al., 2013), yielding inconsistent findings. For declarative knowledge, Lui et al. (2020) showed that IVR environments significantly outperformed DVR in learning scientific concepts. In contrast, Richards and Taylor (2015) compared traditional classroom teaching with 2D and 3D model simulations for learning the biological principle, finding that 2D models were more effective, presumably due to additional cognitive load imposed by 3D models. Differing from both, Kozhevnikov et al. (2013) found that both IVR and DVR facilitated the understanding of relative motion concepts, but the IVR group performed better than the DVR group in solving two-dimensional problems, indicating that IVR is more conducive to transfer of physics learning content. For procedural knowledge, in contrast to the findings of Buttussi and Chittaro (2018), Lovreglio et al. (2021) demonstrated that VR-based simulation training in fire-extinguisher operation significantly improved both knowledge acquisition and long-term retention among trainees. This is primarily because IVR provides a safe and repeatable virtual practice environment, enabling users to repeatedly practice and master skills in a risk-free setting, promoting skill automation and situational adaptation. It is particularly suitable for training with high difficulty (Combalia et al., 2024), high risk, high sensitivity, and low frequency in real-world scenarios (Wilkerson et al., 2008; Karabiyik et al., 2019). As a systematic review has pointed out, IVR is most commonly used for teaching procedural knowledge (Radianti et al., 2020).

Although Parong and Mayer (2018) conducted knowledge classification, they compared factual knowledge and conceptual knowledge, both of which belong to declarative knowledge. They found that IVR was inferior to PowerPoint in factual knowledge acquisition, but there was no significant difference between IVR and PowerPoint in conceptual knowledge acquisition. Although one study has examined the differential effects of IVR and video-based instruction on declarative versus procedural knowledge (Makransky et al., 2021), it did not fully disentangle the independent contributions of immersion and interactivity. Therefore, the present study manipulates immersion by varying the visual field of view in the learning materials and simultaneously investigates the acquisition of declarative and procedural knowledge under different immersion levels. Given that most prior research has demonstrated positive effects of high-immersion environments on both types of knowledge, we hypothesize that:

**Hypothesis 1 (H1).**  *Learning outcomes for both declarative and procedural knowledge will be significantly better under IVR than under DVR.*

### 2.3. Cognitive and Affective Factors of Immersive Learning

The Cognitive Affective Model of Immersive Learning (CAMIL) describes how immersion influences affective and cognitive factors such as learners’ motivation, self-efficacy, cognitive load, and self-regulation by affecting presence and agency, ultimately impacting knowledge acquisition and transfer (Makransky & Petersen, 2021). Several studies have tested and revised CAMIL (Petersen et al., 2022; Makransky & Mayer, 2022). Petersen et al. (2022) validated and adjusted CAMIL through research, categorizing technical factors into immersion and interactivity, and refining elements such as cognitive load and motivation. Makransky and Mayer (2022) further simplified the technical and cognitive-affective factors of the CAMIL model, classifying them into immersive HMD and 2D video based on immersion levels, while subdividing the dimensions of learning outcomes.

According to the CAMIL model, immersion is a key factor in IVR learning environments, influencing learners’ cognitive processing and affective engagement. In the affective domain, this study examines presence, intrinsic motivation, and self-efficacy. Presence is defined as the subjective sensation of “being there” (Slater & Wilbur, 1997), potent enough to override the awareness that the environment is virtual. Numerous studies have demonstrated that IVR elicits significantly higher levels of presence than less-immersive media (Klingenberg et al., 2024; Makransky & Mayer, 2022; K. Kim et al., 2014; Calvert & Abadia, 2020; Makransky & Lilleholt, 2018). Intrinsic motivation refers to the active engagement in an activity driven by its inherent interest, challenge, or sense of accomplishment, rather than by any external outcome (Makransky & Petersen, 2021). Cross-media studies (Makransky & Lilleholt, 2018; Parong & Mayer, 2018; Liu et al., 2022; Elena et al., 2018) on immersion consistently demonstrate that IVR-based learning elicits higher levels of interest, enjoyment, and participation, thereby enhancing intrinsic motivation. Self-efficacy denotes individuals’ subjective belief in their capability to learn or to perform a given task. Empirical work has further confirmed that IVR-based learning positively influences self-efficacy (Buttussi & Chittaro, 2018; Klingenberg et al., 2020; Makransky et al., 2021), with heightened interactivity and sense of control yielding even stronger efficacy beliefs. Accordingly, we propose the following hypotheses:

**Hypothesis 2 (H2).**  *For the learning of both declarative and procedural knowledge, enhancing immersion will increase learners’ presence.*

**Hypothesis 3 (H3).**  *For the learning of both declarative and procedural knowledge, enhancing immersion will strengthen learners’ intrinsic motivation.*

**Hypothesis 4 (H4).**  *For the learning of both declarative and procedural knowledge, enhancing immersion will improve learners’ self-efficacy.*

On the cognitive side, this study examines cognitive load and self-regulation. Cognitive load is ascribed to the cognitive demands imposed by the learning task. Multiple studies indicate that highly immersive environments can increase extraneous cognitive load (Parong & Mayer, 2018; Makransky et al., 2019; Richards & Taylor, 2015; Moreno & Mayer, 2004), underscoring the need for careful attention to load-related design features in IVR-based instruction. Self-regulated learning (SRL) originates in the mind as a sequence of metacognitive episodes that encompass planning, monitoring, evaluation, and adjustment (Zimmerman, 2002). By affording high social presence, IVR enables learners to interact naturally with objects, agents, and information in the virtual environment (Makransky & Petersen, 2021), thereby fostering SRL. One study (Sobocinski et al., 2023). that captured and measured SRL processes within IVR revealed that elevated cognitive load can trigger metacognitive monitoring, which in turn elicits behavioral adjustments. Accordingly, we hypothesize:

**Hypothesis 5 (H5).**  *For the learning of both declarative and procedural knowledge, enhancing immersion will increase learners’ cognitive load.*

**Hypothesis 6 (H6).**  *For the learning of both declarative and procedural knowledge, enhancing immersion will promote learners’ self-regulated learning.*

Although the CAMIL model summarizes the impact of immersion on cognitive-affective factors and distinguishes between factual, conceptual, and procedural knowledge, only a few studies have tested its hypotheses and analyzed these constructs, and emotional factors—which have important implications for cognition—have not been incorporated. Compared with DVR, studies have shown that IVR is more effective at eliciting learners’ pleasant experiences and positive affect (Makransky & Lilleholt, 2018; Elena et al., 2018), which in turn exerts a significant positive influence on knowledge acquisition. Accordingly, we hypothesize:

**Hypothesis 7 (H7).**  *For the learning of both declarative and procedural knowledge, enhancing immersion will elicit learners’ positive emotions.*

## 3. Materials and Method

### 3.1. Research Design

This study adopted a 2 × 2 mixed factorial design, with immersion levels (high immersion and low immersion) as the between-subjects variable and knowledge types (declarative knowledge and procedural knowledge) as the within-subjects variable, consisting of two experimental groups. It investigated learners’ affective experiences, cognitive performance, and learning outcomes when learning the two types of knowledge under two different immersion environments.

### 3.2. Participants

Owing to the need for VR headsets and a specialized laboratory, we adopted a convenience-sampling strategy with experimental subsidies and recruited 85 student participants from a university in Tianjin, China, through public advertisements. Before the official start of the experiment, participants completed a pre-test questionnaire to assess their familiarity with the learning content. Twenty-one participants with excessively high prior knowledge levels were excluded, resulting in 64 valid samples (Mage = 22.13, SD = 1.91). The minimum sample size necessary for this research was established using G*Power 3.1 to guarantee the statistical significance of the findings (Faul et al., 2009). Referring to the effect size d = 0.7 reported in the previous literature (Makransky & Mayer, 2022), and setting the statistical power at 0.80 and the alpha level at 0.05, the results indicated that the minimum required sample size was 52 participants. The actual sample size recruited for this study was 64 participants, which met the requirements for statistical analysis. The demographic characteristics of these participants are presented in Table 1. Among them, there were 43 females and 21 males, with academic backgrounds in science/engineering and humanities/social sciences, excluding biology and medicine. All learners were native Chinese speakers with normal or corrected visual and auditory acuity, no prior VR experience, and no familiarity with the relevant learning content. Participants were randomly assigned to the high immersion group (*n* = 32) and low immersion group (*n* = 32). The χ2 test and Fisher’s exact test were conducted to assess the equivalence of conditions on demographic variables. These were all non-significant: Educational level (*p* = 0.637), gender (*p* = 1.000), and Major (*p* = 1.000). Therefore, we conclude that the assigned groups did not significantly differ based on demography. Based on the principle of voluntary participation, all participants signed informed consent forms before the experiment and received compensation after completion.

### 3.3. Learning Materials

**Figure 1:**
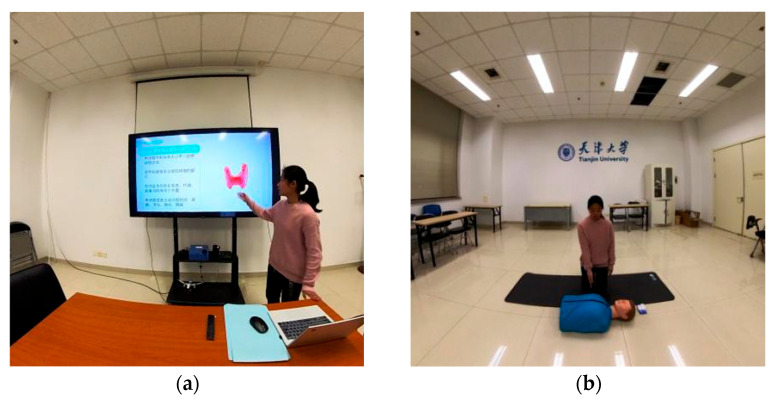
Learning materials. (**a**) Declarative Knowledge; (**b**) Procedural Knowledge.

The learning materials used in the experiment were developed by researchers based on the nature of the two types of knowledge. “Thyroid and related diseases” served as the declarative knowledge topic, covering the morphological structure of the thyroid gland, anabolic metabolism of thyroid hormones, physiological functions, and related diseases. Learners were required to understand and memorize a large amount of factual information and complex biological concepts, with a learning duration of 5 min and 19 s, as shown in Figure 1a. “Cardiopulmonary resuscitation (CPR) procedures” served as the procedural knowledge topic, consisting of eight key steps of CPR and their operational key points. Learners needed to not only master theoretical knowledge but also apply the learned skills in practice, with a learning duration of 5 min and 19 s, as shown in Figure 1b. Qualified professionals were invited as instructors to deliver the thyroid content and demonstrate CPR operations.

### 3.4. Setting and Apparatus

**Figure 2:**
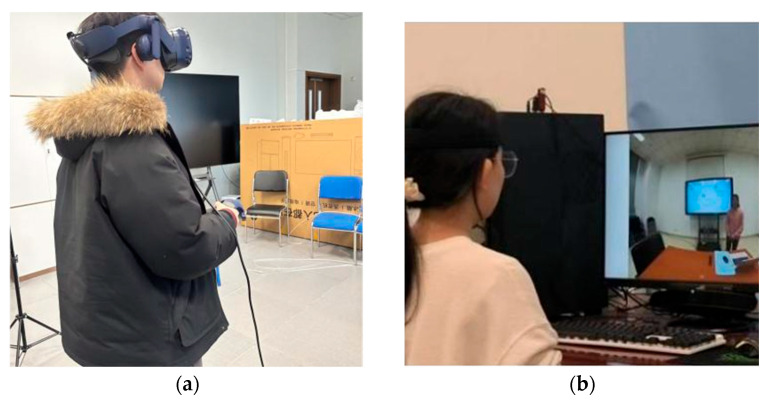
Two different experimental conditions. (**a**) High-immersive environment; (**b**) Low-immersive environment.

According to the experimental design, we created two levels of immersion environments (high immersion vs. low immersion). After filming the teaching scene with an Insta 360 camera(Insta360, Arashi Vision Inc., Shenzhen, China), the videos were converted into 360° panoramic and 2D flat formats using Insta 360 Studio v5.7.2. Participants viewed 360° videos from a first-person perspective using a Head-Mounted Display (HMD, HTC Vive Pro headset; HTC Corporation, New Taipei City, Taiwan) with Viveport Video 4.4.5 (an immersive video player developed by HTC VIVEPORT), achieving a high-immersion experience, as shown in Figure 2a. Low-immersion experience was achieved by viewing 2D videos on a desktop computer screen, as shown in Figure 2b. During video viewing, participants were not required to perform human–computer interaction operations. This experimental treatment effectively excluded other factors that might affect the results, such as interactivity, learning content, and instructor differences.

### 3.5. Measurement

#### 3.5.1. Basic Information and Prior Knowledge Questionnaire

The basic information questionnaire collected participants’ demographic characteristics, including age, gender, and major. The prior knowledge test assessed participants’ familiarity with the learning content, with 5 questions for each type of knowledge, using a 5-point Likert scale ranging from “very unfamiliar (1 point)” to “very familiar (5 points)”.

#### 3.5.2. Learning Outcome Tests

Learning outcomes were measured using knowledge tests. The knowledge tests were designed by the research team based on existing test questions on “thyroid and related diseases” and “CPR procedures” combined with the knowledge points of this study, and were reviewed and approved by a professor specializing in medicine. The declarative knowledge test assessed learners’ knowledge mastery, consisting of 13 questions, 1 point for each question, with a total of 13 points. The procedural knowledge test evaluated learners’ mastery of skill operation key points, with 25 rating items based on operational key points, 1 point for each item, with a total of 25 points. The scoring of skill tests was independently completed by two trained researchers, and Pearson correlation analysis showed high inter-rater reliability (r = 0.95).

#### 3.5.3. Scales

Presence Scale: Modified from the method of T. Kim and Biocca (1997), with higher total scores indicating stronger presence. It demonstrated good reliability (Cronbach’s α = 0.777).

Positive Affect Scale: Adapted from the Positive and Negative Affect Schedule (PANAS) Emotional Scale (Watson & Clark, 1994), consisting of 9 items. Higher scores indicate stronger perceived positive emotions, with high reliability (Cronbach’s α = 0.907).

Self-Efficacy Scale: Adapted from the measurement tool developed by Artino and McCoach (2008), consisting of 5 items. Higher scores represent stronger self-efficacy, showing high reliability (Cronbach’s α = 0.870).

Self-Regulation Scale: Adopted the tool developed by Broadbent et al. (2023), which assesses learners’ use of metacognitive strategies. It consists of 5 items with good reliability (Cronbach’s α = 0.792).

The above Presence, Affect, Self-Efficacy, and Self-Regulation scales were all measured using a 5-point Likert scale, where “1” indicates “strongly disagree” and “5” indicates “strongly agree”.

Intrinsic Motivation Scale: Based on the version developed by Isen and Reeve (2005), using a 7-point continuous Likert scale (from “1 = strongly disagree” to “7 = strongly agree”). The total score of the 9 items reflects the strength of learners’ intrinsic motivation, with good reliability (Cronbach’s α = 0.730).

Cognitive Load Scale: Developed with reference to Paas (1992)’s measurement tool, using a 9-point Likert scale. It includes three dimensions, with one item per dimension, and has good reliability (Cronbach’s α = 0.837).

### 3.6. Procedure

Each participant entered the laboratory individually. The experimenter first explained the experimental procedure, task requirements, and precautions. After participants read and signed the Experimental Informed Consent Form, they completed a questionnaire containing basic information (age, gender, major, etc.) and self-assessment scales for CPR and thyroid knowledge. Participants were then randomly assigned to one of the two immersion environments, adapted to the environment, and confirmed the volume by watching a video unrelated to the learning content. They subsequently engaged in the two types of knowledge learning in random order (after each type of learning, participants completed a learning effectiveness test and a learning experience questionnaire), with a 10 min break scheduled between sessions. After the experiment, participants received compensation for their participation and were thanked.

## 4. Results

### 4.1. Differences in Learning Outcomes Between Groups

**Figure 3:**
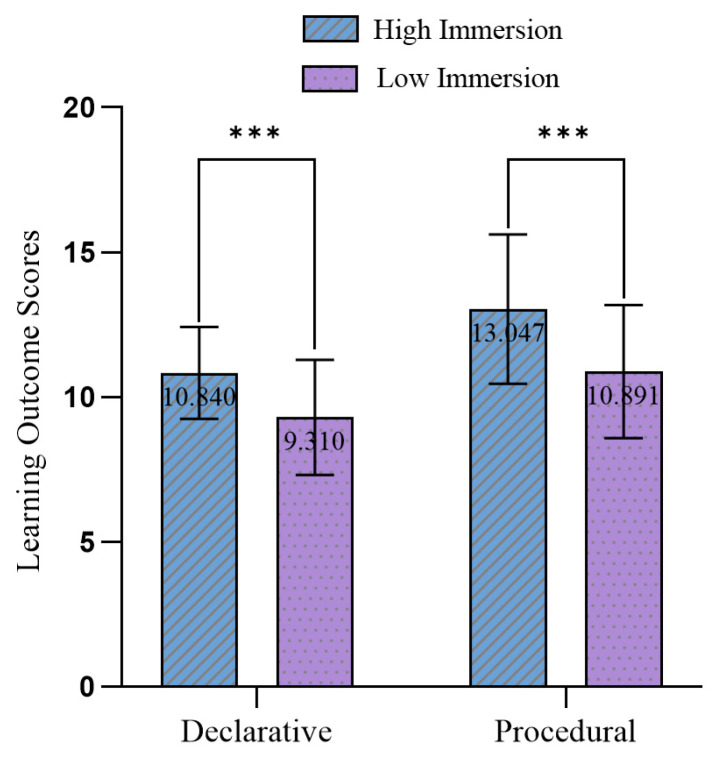
Scores of declarative and procedural knowledge tests for two groups (error bars represent standard deviation). *** *p* < 0.001.

The results of the knowledge test scores for the high-immersion and low-immersion groups are shown in Figure 3. To examine whether there were significant differences in the learning outcomes of two types of knowledge under different immersion levels, this study employed an independent samples t-test for statistical analysis. Table 2 shows that there was a significant difference in the impact of different immersion levels on declarative knowledge learning outcomes, with the high immersion group scoring higher (M = 10.84, SD = 1.59) than the low immersion group (M = 9.31, SD = 1.99), *t*(62) = 3.401, *p* < 0.05. Since the mean difference (MD) was 1.53 and the pooled standard deviation was approximately 1.79, d = 0.85 (95% CI [0.33, 1.35]), a value that indicates a large effect size according to Cohen (1988). Similarly, there was a significant difference in the impact of immersion levels on procedural knowledge learning outcomes, with the high immersion group scoring higher (M = 13.05, SD = 2.58) than the low immersion group (M = 10.89, SD = 2.30), *t*(62) = 3.530, *p* < 0.05. Since the mean difference (MD) was 2.16 and the pooled standard deviation was approximately 2.44, d = 0.89 (95% CI [0.37, 1.39]), a value that indicates a large effect size according to Cohen (1988). Therefore, Hypothesis H1 was supported.

### 4.2. Differences in Cognitive and Affective Factors Between Groups

**Figure 4:**
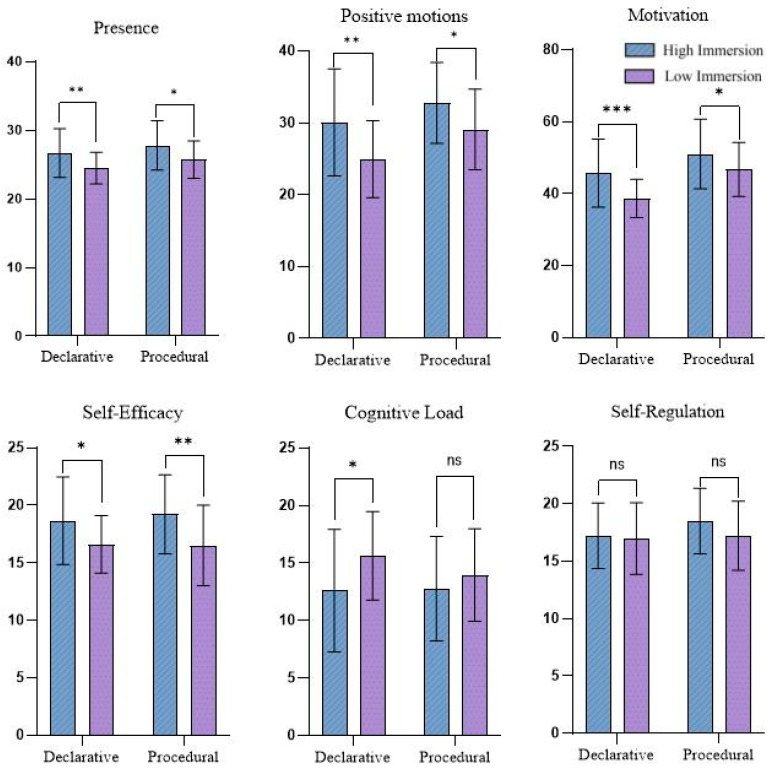
Scores on cognitive and affective factors for the two groups. (Error bars represent standard deviation.) * *p* < 0.05. ** *p* < 0.01. *** *p* < 0.001, ‘ns’ indicates non-significance (*p* > 0.05).

The scores of cognitive and affective factors for the high-immersion and low-immersion groups are presented in Figure 4. Independent *t*-tests were conducted to analyze the impact of different immersion levels (high immersion vs. low immersion) on learners’ cognitive and affective factors, with results presented in Table 3.

Presence. In declarative knowledge learning, the presence score of the high immersion group (M = 26.69, SD = 3.54) was significantly higher than that of the low immersion group (M = 24.47, SD = 2.30), *t*(62) = 2.972, *p* < 0.01, d = 0.75 (95% CI [0.23, 1.35]). In procedural knowledge learning, the presence score of the high immersion group (M = 27.78, SD = 3.61) was also significantly higher than that of the low immersion group (M = 25.72, SD = 2.73), *t*(62) = 2.58, *p* < 0.05, d = 0.65 (95% CI [0.14, 1.15]). Hypothesis H2 (enhanced immersion improves learners’ presence) was supported.

Motivation. In declarative knowledge learning, the intrinsic motivation score of the high immersion group (M = 45.72, SD = 9.48) was significantly higher than that of the low immersion group (M = 38.66, SD = 5.33), *t*(62) = 3.674, *p* < 0.001, d = 0.92 (95% CI [0.40, 1.43]). In procedural knowledge learning, the intrinsic motivation score of the high immersion group (M = 51.03, SD = 9.67) was significantly higher than that of the low immersion group (M = 46.70, SD = 7.51), *t*(62) = 2.006, *p* < 0.05, d = 0.50 (95% CI [0.002, 0.997]). Hypothesis H3 (enhanced immersion increases intrinsic motivation) was supported.

Self-Efficacy. In declarative knowledge learning, the self-efficacy score of the high immersion group (M = 18.66, SD = 3.82) was significantly higher than that of the low immersion group (M = 16.59, SD = 2.49), *t*(62) = 2.558, *p* < 0.05, d = 0.64 (95% CI [0.13, 1.14]). In procedural knowledge learning, the self-efficacy score of the high immersion group (M = 19.22, SD = 3.43) was significantly higher than that of the low immersion group (M = 16.50, SD = 3.49), *t*(62) = 3.14, *p* < 0.05, d = 0.79 (95% CI [0.27, 1.29]). Hypothesis H4 (enhanced immersion improves self-efficacy) was supported.

Cognitive Load. In declarative knowledge learning, the cognitive load score of the high immersion group (M = 12.59, SD = 5.33) was significantly lower than that of the low immersion group (M = 15.63, SD = 3.84), *t*(62) = −2.609, *p* < 0.05, d = 0.65 (95% CI [0.18, 1.15]). In procedural knowledge learning, there was no significant difference in cognitive load between the two groups (M = 12.75 vs. 13.94), *t* = −1.11, *p* > 0.05, d = −0.28 (95% CI [0.00, 0.75]). Hypothesis H5 (enhanced immersion increases cognitive load) was not fully supported.

Self-Regulation. In declarative knowledge learning, there was no significant difference in self-regulation scores between the high immersion group (M = 17.22, SD = 2.84) and the low immersion group (M = 16.97, SD = 3.11), *t*(62) = 0.336, *p* > 0.05, d = 0.08 (95% CI [−0.40, 0.57]). In procedural knowledge learning, there was also no significant difference in self-regulation scores between the high immersion group (M = 18.50, SD = 2.85) and the low immersion group (M = 17.22, SD = 3.01), *t*(62) = 1.747, *p* > 0.05, d = 0.44 (95% CI [−0.06, 0.93]). Hypothesis H6 (enhanced immersion promotes self-regulation) was not supported.

Positive Emotions. In declarative knowledge learning, the positive emotions score of the high immersion group (M = 30.06, SD = 7.43) was significantly higher than that of the low immersion group (M = 24.94, SD = 5.35), *t*(62) = 3.167, *p* < 0.05, d = 0.79 (95% CI [0.28, 1.30]). In procedural knowledge learning, the positive emotions score of the high immersion group (M = 32.78, SD = 5.62) was significantly higher than that of the low immersion group (M = 29.09, SD = 5.61), *t*(62) = 2.626, *p* < 0.05, d = 0.66 (95% CI [0.15, 1.16]). Hypothesis H7 (enhanced immersion elicits positive emotions) was supported.

## 5. Discussion

This study preliminarily investigated differences in learning outcomes for different types of knowledge under high and low immersion environments. The results showed that for both types of knowledge, enhanced immersion significantly improved learning effectiveness and affective experiences but did not significantly promote self-regulation during the learning process. Our findings also indicated that enhanced immersion does not always significantly optimize learners’ cognitive load levels, as its effects are related to knowledge types. In the subsequent discussion section, we will focus on analyzing the reasons for the differences in learning effectiveness between declarative and procedural knowledge.

### 5.1. Enhanced Immersion Significantly Improves Learning Effectiveness for Both Declarative and Procedural Knowledge

This study found that enhanced immersion has a significant positive effect on declarative knowledge learning, which is consistent with previous research (Calvert & Abadia, 2020; Makransky & Mayer, 2022). A possible reason is that the “sense of isolation” created by immersive VR could effectively reduce the risk of distraction, prompting learners’ visual and auditory senses to be fully immersed in the learning content, stimulating deep thinking and active exploration (Carassa et al., 2002), and promoting cognitive engagement and information processing to construct mental representations of learning materials and experiences (Makransky et al., 2019), ultimately resulting in learning outcomes more conducive to abstract knowledge transfer. However, relevant studies (Klingenberg et al., 2020; Makransky et al., 2021) found that mere immersion (IVR vs. DVR comparison) had no significant positive impact on learning effectiveness, but when combined with generative learning strategies, IVR was more effective than DVR in improving transfer ability and knowledge retention. The “Method Effects on Learning Hypothesis” (Moreno, 2006) may serve as a possible explanation. The CAMIL (Makransky & Petersen, 2021) model also points out that there is an interaction effect between media and method. In IVR-based learning, certain instructional approaches may render IVR a more effective learning tool precisely because they can fully harness its high immersion and high interactivity. The immersion principle in multimedia learning (Makransky, 2021) further emphasizes that the key to enhancing learning effectiveness does not lie in immersion itself, but in instructional design that aligns with the characteristics of immersion.

The results also indicated that enhanced immersion could further improve skill learning effectiveness for procedural knowledge, consistent with previous research findings. Alhalabi (2016) compared the impact of three VR systems and traditional methods on student performance, finding that VR-based learning significantly outperformed non-VR groups in two skill tests (mathematical operations and graphic analysis), with the IVR system (highest immersion) yielding the highest average student test scores. The reasons may be explained by the learning characteristics of operational skills and the unique role of high-immersion environments. Operational skill learning is often situational and practical, requiring learners to fully perceive the environment and movements, and then master them through repeated practice. High-immersion environments, through panoramic views, allow learners to feel as if they are in a real operational scenario. The embodied 3D visualization and 360-degree full-field coverage not only provide multi-dimensional visual information, enabling learners to perceive more details, material context, and semantic organizational structures (Plancher et al., 2012), but also support observing operational processes from different angles, facilitating learners’ mental rotation, special representation, and memory of movement information (Araiza-Alba et al., 2021). High-immersion environments represented by IVR could provide multiple depth cues not available in other technologies (Bowman & Mcmahan, 2007), helping learners improve the speed, accuracy, and comprehension of spatial memory (Schuchardt & Bowman, 2007), and promoting the mastery of operational steps and key points.

Although the present results indicate that IVR can enhance learning effectiveness, several practical factors must be considered when the technology is transferred to real educational settings. Because DVR requires no dedicated experiential zone, can be launched immediately, and supports simultaneous whole-class learning, it retains clear advantages in contexts where space, equipment, or scalability are constrained. Educators should therefore weigh IVR’s high immersion against DVR’s greater convenience in light of specific instructional goals, available resources, and practical limitations to achieve the optimal balance.

### 5.2. Enhanced Immersion Significantly Improves Affective Experiences but Has Limited Impact on Cognition

This study found that during the learning of both types of knowledge, enhanced immersion significantly improved learners’ presence, self-efficacy, intrinsic motivation, and positive emotions. The findings on presence are consistent with most existing research (Calvert & Abadia, 2020; Klingenberg et al., 2024), indicating that the “first-person egocentric experience” in high-immersion environments may more effectively enhance learners’ presence (Kozhevnikov et al., 2013). The conclusions related to emotions are similar to most research results (Parong & Mayer, 2021; Elena et al., 2018), suggesting that high-immersion learning experiences increase learning pleasure through rich sensory stimulation, evoke positive emotions, and enhance learners’ engagement, confidence, and energy levels. A strong sense of presence and positive emotion can significantly enhance the depth of memory processing. Through the encoding specificity principle (Tulving & Thomson, 1973) and the emotion-enhanced memory effect (LaBar & Cabeza, 2006), information becomes easier to recall during retrieval due to the reappearance of contextual cues, thereby improving perceived learning outcomes. The findings on intrinsic motivation are consistent with the research by Makransky and Lilleholt (2018). The realistic characteristics and good affordances of IVR learning environments may make students perceive the fun, value, and significance of learning content, recognize the cognitive benefits of learning, and thus become more motivated to learn. Regarding self-efficacy, the results are consistent with Klingenberg et al. (2020), who found that immersive VR leads to higher self-efficacy. In high-immersion environments, learners experience increased focus, enhanced sense of control, altered time perception, and diminished self-awareness (Freude et al., 2020), fully focusing on current learning tasks. Learners in a state of flow could more efficiently mobilize cognitive resources, skills, and strategies to complete tasks and overcome challenges, thereby accumulating direct experiences of success and improving self-efficacy.

However, neither for the acquisition of declarative knowledge nor for the mastery of procedural knowledge did the high-immersion environment produce a significant facilitation of learners’ self-regulatory processes. A plausible explanation is that the IVR’s high immersion may direct substantial cognitive resources to the processing of external stimuli, leaving insufficient capacity to support simultaneous, higher-order metacognitive reflection (Sweller et al., 2019) and impairing the monitoring of internal cognitive states. Moreover, the high presence induced by IVR may make it difficult for learners to disengage from the scenario and adopt an outsider’s perspective to inspect their affective, cognitive, metacognitive and motivational processes, unless adequate scaffolds are provided (Makransky & Petersen, 2021). Thus, embedding reflective activities that foster metacognition and deep learning during or immediately after IVR is crucial for effective self-regulated learning. The conclusion is inconsistent with the findings of Sobocinski et al. (2023), which would be due to differences in measurement methods: Sobocinski’s study used qualitative coding to analyze think-aloud audio to investigate self-regulation behaviors in IVR environments, whereas the scale tests used in this study rely on learner self-reports, which are susceptible to memory biases and state fluctuations, making it difficult to accurately reflect the metacognitive activities of regulatory behaviors.

Meanwhile, the enhanced immersion generated a differentiated effect on cognitive load between the two knowledge types, thereby revealing that the high-immersion environment’s capacity to optimize overall cognitive load is relatively limited. For declarative knowledge, enhanced immersion significantly reduced learners’ cognitive load, which is consistent with the findings of Liu et al. (2022). When learners are immersed in an IVR environment, they could focus more on learning content, reduce external distractions, concentrate cognitive resources on learning, and promote generative processing (Makransky & Lilleholt, 2018), thereby reducing extraneous cognitive load caused by distraction. However, this result differs from some studies (Richards & Taylor, 2015; Parong & Mayer, 2018); for example, Makransky et al. (2019) found via electroencephalogram (EEG) measurements that IVR induces higher cognitive load than DVR. This discrepancy may be due to the stricter control of VR media characteristics in this study: interactive operations of IVR systems are the main factor causing extraneous cognitive load (Sobocinski et al., 2023), and this study completely separated immersion from other technical features, avoiding interference from human–computer interaction factors, which may have yielded more reliable results.

In contrast, enhanced immersion did not significantly reduce cognitive load in procedural knowledge learning, possibly due to three reasons. First, IVR-specific sources of extraneous cognitive load—visual-sensory demands, and potential discomfort—are absent in DVR, yet they add to learners’ cognitive burden. Among these, VR sickness is a common side effect of IVR; users of head-mounted displays frequently report headaches, dizziness, and similar symptoms (Woo et al., 2023) produced by vestibular-visual conflict. According to the limited-resources model of cognition (Kahneman, 1973), the brain must allocate working-memory capacity to compensate for vestibular instability. This process inevitably consumes cognitive resources that would otherwise be devoted to learning tasks, reducing the share available for comprehending, memorizing, and sequencing procedural knowledge. Second, differences in both learning materials and processing mechanisms between the two knowledge types may jointly attenuate the cognitive benefits of high-immersion environments. At the material-design level, the learning materials for the two types of knowledge were inconsistent, with differences in content complexity and information organization, leading to varying information volumes in the learning environment and thus different impacts on cognitive load. At the processing-mechanism level, the two knowledge forms rely on distinct learning processes that impose qualitatively and quantitatively different working-memory requirements. Declarative learning emphasizes semantic-network construction, whereas procedural tasks require the integration of motor-visual feedback. Because automaticity of operational steps must be maintained, procedural knowledge demands continuous working-memory engagement and higher overall cognitive resources (Hong et al., 2018), limiting the reduction in cognitive load due to skill maintenance requirements. Third, the novelty effect of IVR offers an additional explanation. Using the new technology may trigger task-irrelevant information processing during learning (Parong & Mayer, 2021), thereby imposing extraneous cognitive load. In this study, the novelty and excitement of a first-time VR experience captured part of the participants’ attention, diverting cognitive resources that would otherwise have been allocated to encoding the operational procedure.

## 6. Conclusions and Limitations

The core contribution of this study lies in integrating a knowledge-classification framework with a controlled-immersion design to simultaneously examine the differential effects of VR immersion on the acquisition of declarative and procedural knowledge. Results show that heightened immersion significantly improves learning outcomes for both knowledge types and enhances learners’ affective experience, yet its facilitative effect on cognitive processing remains limited. These findings provide new empirical support for cognitive-affective models of immersive learning, design principles for reducing extraneous cognitive processing in multimedia learning, and the method-affects-learning hypothesis, while offering practical insights for optimizing VR-based immersive learning.

Educators should balance immersion intensity with knowledge-type fit during material design, eliminate redundant audio-visual stimuli, and include affective and cognitive process indicators in learning-outcome assessments. Researchers and developers ought to follow multimedia instructional design principles, using scaffolds, embedded tests, and question prompts to reduce extraneous processing, and couple VR with self-regulated learning (SRL) and problem-based learning (PBL) to foster deep conceptual understanding, transfer, and meaningful learning. Meanwhile, they need to guard against technological obsolescence (Vergara et al., 2019). For instance, the HTC VIVE VR hardware and software, as well as the VR cameras used in this study, are likely to become outdated as technology advances. Newer VR devices may offer enhanced immersion experiences. Therefore, while researchers and developers should stay attuned to technological trends, it is crucial to focus on the pedagogical principles underlying immersion.

There are also several limitations in this study that need to be addressed in future research. First, we failed to achieve a balance in difficulty between the two knowledge types. Owing to differences in content complexity and information structure, declarative and procedural tasks may vary in information load, thereby eliciting differential cognitive load and self-regulatory demands. Future research should manipulate the information volume of learning materials to match the material’s difficulty before examining whether immersion and knowledge type interact. Furthermore, control group designs might be used to analyze content difficulty as an independent variable, isolating the impact of content differences. Second, this study used tests and scales to collect quantitative data and examined the differential effects of immersion on learning outcomes. Constrained by sample size, the mechanisms underlying these differences were not explored—for example, by constructing a structural equation model to test mediating or moderating effects. Future research could enlarge the sample and incorporate mediating and moderating variables to systematically unpack the pathways through which immersion influences learning outcomes. In addition, objective measures such as EEG and fNIRS could be integrated to deepen our understanding of the underlying cognitive and affective processes mechanisms at play during learning. Third, the sample over-represented graduate students (70.3%) and consisted of novices from a single university, yielding high homogeneity in age, cultural, and technology familiarity. Consequently, the findings primarily apply to higher-education learners with low prior knowledge, yet an expertise reversal effect cannot be ruled out (Magner et al., 2014). It should also be noted that participants generally lacked prior VR experience, rendering them susceptible to a novelty effect that may confound the evaluation of learning outcomes. Future studies should employ multi-site, stratified sampling across educational stages and expertise levels to verify the robustness of the observed effects.

## Figures and Tables

**Table 1:** Participant information.

Variable	Category	Count	Percent (%)
Gender	Male	21	32.8
	Female	43	67.2
Educational level	Undergraduate	19	29.7
	Master’s student	42	65.6
	Doctorate student	3	4.7
Major category	Science, Technology, Engineering, and Mathematics (STEM)	35	54.7
	Humanities and Social Sciences (HSS)	29	45.3

N = 64.

**Table 2:** Results of independent samples *t*-test on learning effects.

Variable	M	SD	*t*	*df*	*p*
Declarative knowledge			3.401	62	0.001 ***
High Immersion	10.84	1.59			
Low Immersion	9.31	1.99			
Procedural knowledge			3.53	62	0.001 ***
High Immersion	13.05	2.58			
Low Immersion	10.89	2.30			

*** *p* < 0.001.

**Table 3:** Results of independent samples *t*-test on cognition and affection.

Knowledge Type	Variable	M	SD	*t*	*df*	*p*
Declarative	Presence			2.972	62	0.004 **
	High Immersion	26.69	3.54			
	Low Immersion	24.47	2.30			
	Positive emotions			3.167	62	0.002 **
	High Immersion	30.06	7.43			
	Low Immersion	24.94	5.35			
	Motivation			3.674	62	0.001 ***
	High Immersion	45.72	9.48			
	Low Immersion	38.66	5.33			
	Self-Efficacy			2.558	62	0.013 *
	High Immersion	18.66	3.82			
	Low Immersion	16.59	2.49			
	Cognitive Load			−2.609	62	0.012 *
	High Immersion	12.59	5.33			
	Low Immersion	15.63	3.84			
	Self-Regulation			0.336	62	0.738
	High Immersion	17.22	2.84			
	Low Immersion	16.97	3.11			
Procedural	Presence			2.58	62	0.012 *
	High Immersion	27.78	3.61			
	Low Immersion	25.72	2.73			
	Positive emotions			2.626	62	0.011 *
	High Immersion	32.78	5.62			
	Low Immersion	29.09	5.61			
	Motivation			2.006	62	0.049 *
	High Immersion	51.03	9.67			
	Low Immersion	46.7	7.51			
	Self-Efficacy			3.14	62	0.003 **
	High Immersion	19.22	3.43			
	Low Immersion	16.5	3.49			
	Cognitive Load			−1.11	62	0.271
	High Immersion	12.75	4.53			
	Low Immersion	13.94	4.02			
	Self-Regulation			1.747	62	0.086
	High Immersion	18.5	2.85			
	Low Immersion	17.22	3.01			

* *p* < 0.05. ** *p* < 0.01. *** *p* < 0.001.

## Data Availability

The data of this study are available from the corresponding author on reasonable request.
